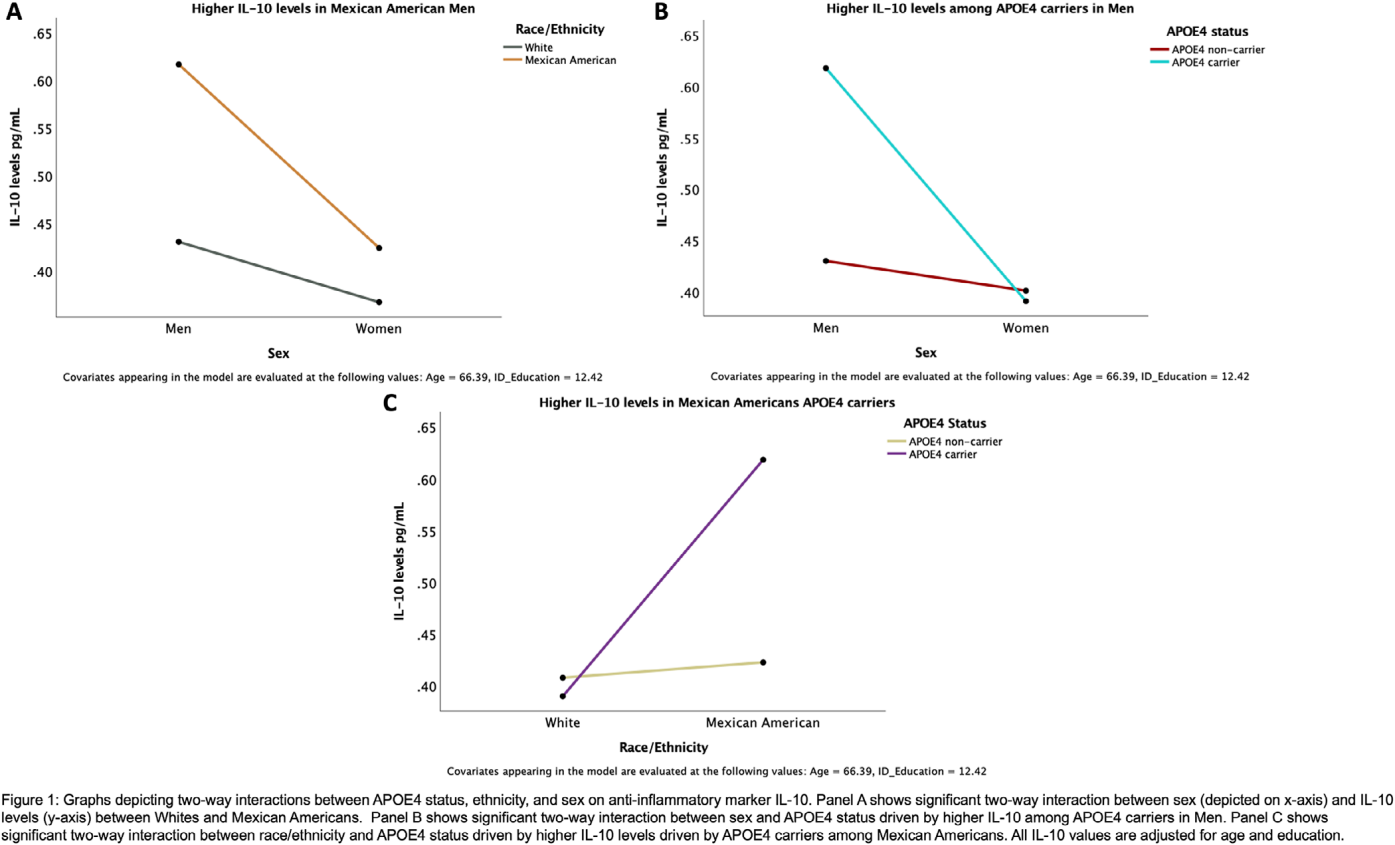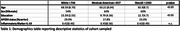# Exploring the Effects of Ethnicity, Sex, and APOE Status on Markers of Inflammation

**DOI:** 10.1002/alz.091862

**Published:** 2025-01-09

**Authors:** Joey A Contreras, Vahan Aslanyan, Nancy E Ortega, Judy Pa

**Affiliations:** ^1^ Alzheimer's Disease Cooperative Study (ADCS), University of California San Diego, La jolla, CA USA; ^2^ Department of Population and Public Health Sciences, Keck School of Medicine, University of Southern California, Los Angeles, CA USA; ^3^ Alzheimer's Disease Cooperative Study (ADCS), University of California San Diego, La Jolla, CA, USA, La Jolla, CA USA; ^4^ Alzheimer's Disease Cooperative Study (ADCS), University of California, San Diego, La Jolla, CA USA

## Abstract

**Background:**

Alzheimer’s Disease (AD) is the most common form of dementia and is considered to disproportionately affect underserved populations like Hispanics/Latinos. Most research available with AD markers such as inflammation and APOE4 has been conducted in predominantly White cohorts, leaving a dearth of knowledge relating APOE4 and inflammation in Hispanic/Latinos. Recent research also suggests inflammation and AD risk may be sex‐specific. This study investigated the effects of race/ethnicity, sex/gender, and APOE4 on inflammatory markers.

**Method:**

Using data from the Health and Aging Brain Study‐Health Disparities (HABS‐HD), we assessed group differences between APOE4 status, ethnicity, and sex on anti‐inflammatory marker interleukin‐10 covarying for age and education.

**Result:**

The dataset included 817 Mexican Americans (65% women, 18% APOE4 carrier, mean age±SD=63.21±8.04 years) and 746 white participants (54% women, 30% APOE4 carrier, mean age±SD=68.59±8.70 years) (Table 1). We found two‐way interactions between sex and race/ethnicity (p=0.03) on IL‐10, driven by higher IL‐10 levels in Mexican American men; sex and APOE4 status (p=0.001), driven by higher IL‐10 among APOE4 carriers in men; and race/ethnicity and APOE4 status (p=0.001), driven by higher IL‐10 levels APOE4 carriers among Mexican Americans (Figure 1A,B and C respectively).

**Conclusion:**

IL‐10 is an important anti‐inflammatory modulator of glial activation, preventing inflammation‐mediated neuronal degeneration under pathological conditions. It has multiple immunoregulatory effects such as in ability to inhibit the production of several inflammatory cytokines. These findings support previous studies demonstrating heightened systemic inflammation in APOE4 carriers, but little has been shown regarding their relationship with gender and ethnicity. This finding suggests the importance of examining differences that may exist between APOE4, sex, and inflammatory markers in Hispanics/Latinos. Future work will examine other inflammatory markers.